# Thermal Stability of Thin Metal Films on GaN Surfaces: Morphology and Nanostructuring

**DOI:** 10.3390/nano15231789

**Published:** 2025-11-27

**Authors:** Andrzej Stafiniak, Wojciech Macherzyński, Adam Szyszka, Radosław Szymon, Mateusz Wośko, Regina Paszkiewicz

**Affiliations:** 1 Faculty of Electronics, Photonics and Microsystems, Wrocław University of Science and Technology, Wybrzeże Wyspiańskiego 27, 50-370 Wrocław, Poland; wojciech.macherzynski@pwr.edu.pl (W.M.); adam.szyszka@pwr.edu.pl (A.S.); mateusz.wosko@pwr.edu.pl (M.W.); regina.paszkiewicz@pwr.edu.pl (R.P.); 2 Department of Experimental Physics, Wrocław University of Science and Technology, Wybrzeże Wyspiańskiego 27, 50-370 Wrocław, Poland; radoslaw.szymon@pwr.edu.pl

**Keywords:** GaN, metal nanostructures, self-assembled nanostructures, SSD, GaN decomposition

## Abstract

The development of metal nanostructures on large-area Gallium Nitride (GaN) surfaces has the potential to enable new, low-cost technologies for III-N semiconductor layer nanostructuring. Self-assembled nanostructures are typically formed through the thermal activation of solid-state dewetting (SSD) in thin metal layers. However, such thermal processing can induce degradation of the metal-GaN material system. This comprehensive study investigated the thermal stability of thin metal films on GaN surfaces, focusing on their morphology and nanostructuring for high-temperature processing. The research expands and systematizes the understanding of the thin metal layers on GaN surface interactions at high temperatures by categorizing metals based on their behaviour: those that exhibit self-assembly, those that catalyze GaN decomposition, and those that remain thermally stable. Depending on the annealing temperature and metal type, varying degrees of GaN layer decomposition were observed, ranging from partial surface modification to significant volumetric degradation of the material. A wide range of metals was investigated: Au, Ag, Pt, Ni, Ru, Mo, Ti, Cr, V, Nb. These materials were selected based on criteria such as high work function and chemical resistance. In this studies metal layers with a target thickness of 10 nm deposited by vacuum evaporation on 2.2 μm thick GaN layers grown by metal organic vapor phase epitaxy were applied. The surface morphology and composition were analyzed using AFM, SEM, EDS, and Raman spectroscopy measurement techniques.

## 1. Introduction

The development of new electronic technologies has enabled the expansion of industrial sectors in branches such as electromobility, 5G telecommunications and renewable energy. However, devices in these fields often encounter material limitations of standard silicon technology owing to higher requirements and more severe operating conditions. Consequently, wide-bandgap materials, for example, AIIIN materials, such as Gallium Nitride (GaN), offer an alternative solution to these challenges.

Due to their high surface-to-volume atom ratio and the quantum effects within the energy band structure of semiconductors, nanostructures exhibit novel physical and chemical properties compared to bulk materials. The nanostructuring of semiconductor substrates can be used in a variety of applications to improve their performance, including, but not limited to, optoelectronic devices (photovoltaic cells, detectors, emitters), electronic devices, gas and biological substance sensors, and energy storage [1,2,3]. The combination of the unique properties of nanostructures with wide-bandgap semiconductors such as AIIIN materials has great application potential for the electronics industry, where the capabilities of devices can be expanded in the power domain, operating frequency, and spectral range, increasing efficiency and sensitivity in both emitting and device-detection applications.

There are two strategies for fabricating nanostructures: a top-down approach, which utilizes wet or dry etching processes of substrates, and a bottom-up approach involving the growth or deposition of nanostructures. Both strategies can exploit the phenomenon of the self-assembly of metallic material [4,5]. This approach reduces production costs by eliminating the need for lithographic processes, which are especially expensive at the nanoscale [6].

The process of self-assembly of thin metallic layers into the form of separate nanoislands on a substrate surface, known as solid-state dewetting (SSD), is driven by the system’s tendency to minimize its total interfacial energy between the GaN substrate and the metallic layer. This phenomenon occurs through the mass transport of thin metal layers to reduce the free energy at the interfaces and achieve the equilibrium shape of the islands [7]. While the mechanisms of the SSD process are well described, most studies have focused on conventional substrates such as Si or SiO_2_/Si [7,8]. However, the results and conclusions obtained in these studies cannot be directly transferred to the metal/AIIIN system, as the substrate structure (its orientation, polarity, roughness, and morphology) greatly influences the course of the SSD process [7,9]. This is particularly critical for surfaces such as heteroepitaxial GaN layers or AlGaN/GaN structures, where defects such as nanocracks, grain boundaries, and surface defects occur [9,10]. Although a few publications have been devoted to the SSD of thin layers, such as Ag, and Au on a GaN surface [11,12], there is a lack of comprehensive studies explaining the mechanisms of nanostructure formation for a wider group of metals. Furthermore, the decomposition of GaN layers at elevated temperatures during interactions with other metals, such as Pt and Pd, is not yet fully understood [9,13].

GaN substrates were grown by metal organic vapor phase epitaxy (MOVPE), a common method for compound semiconductor crystallization. This technique offers significant advantages over other epitaxial methods, providing excellent process control, due to its gaseous precursors, while simultaneously offering scalability and mass production capabilities. Its material versatility, combined with the unique ability to control growth from the atomic layer level up to multi-micrometer-thick structures, makes MfigOVPE the preferred method for manufacturing a wide range of nano- and microelectronic devices. In GaN technology, MOVPE represents the state-of-the-art production technique. The results of our previous work on group III-nitride epitaxy can be found in [14,15,16].

Our research focused on the thermal stability of thin metal films on GaN surfaces in terms of morphology and microstructure, expanding knowledge of the SSD mechanism of metal layers on the GaN surface. We determined which metals exhibit self-assembly, which catalyze GaN decomposition, and which remain thermally stable on the GaN surface. Depending on the annealing temperature and the specific metal type, varying degrees of GaN layer decomposition were observed, ranging from partial surface modification to significant volumetric degradation of the material.

The knowledge acquired on the possibility of producing self-assembled metal patterns on large-format GaN layer surfaces will lead to the development of new low-cost technologies for AIIIN layer nanostructuring using wet or dry etching techniques. Metallic nanostructures on GaN surface can serve as a mask in reactive ion etching (RIE) or as a catalyst in wet metal-assisted chemical etching (MaCE) processes [4,17,18,19,20,21,22]. The degradation of GaN layers due to metal interaction can be mitigated by the use of dielectric capping layers such as SiO_2_ [9,18,19]. However, this reduces the efficiency of RIE processes and limits the applicability of MaCE. Furthermore, metals that exhibit thermal stability on the GaN surface can be employed as masking layers in high-temperature processing or as electrode materials in various devices.

## 2. Materials and Methods

In the studies, 2.2 µm thick GaN layers grown by MOVPE using AIXTRON CCS FT 3 × 2′′ R&D (Aachen, Germany) system on 2′′ c-plane (0001) sapphire substrates were utilized. The growth was performed using trimethylgallium (TMGa, Marburg, Germany), ammonia (NH_3_, 7N, Messer, Bad Soden am Taunus, Germany) precursors, with H_2_ (7N) as the carrier gas. The process followed a standard five-stage procedure: 1—annealing of sapphire substrate in a hydrogen atmosphere; 2—substrate nitridation in H_2_ and NH_3_ mixture; 3—low-temperature growth of GaN nucleation layer; 4—ramping to a temperature which promotes coalescent growth of the high-temperature (HT) GaN buffer layer; 5—growth of the final HT-GaN buffer layer. The substrates were subsequently diced into 10 × 10 mm samples. For comparative analysis, 10 × 10 mm Si samples with a 200 nm SiO_2_ layer deposited by plasma-enhanced chemical vapor deposition were also prepared (PECVD, PlasmaLab80+ Oxford Instruments, Abingdon, UK).

In the next step, thin metal layers were deposited on the samples using the physical vapor deposition method by resistive thermal (Au, Ag) or electron beam (Pt, Ni, Ru, Mo, Ti, Cr, V, Nb) evaporation technique (UHV PVD225, Kurt J. Lesker, Jefferson Hills, PA, USA). The target thickness for all metal layers was 10 nm. After the evaporation process, the thickness of the layers was measured using the white-light interferometer (Talysurf CCI, Taylor-Hobson, Leicester, UK). Table 1 summarizes the metals used, their measured film thicknesses, deposition rates, and relevant physical properties.

After deposition of the metal layers, the samples were annealed in a nitrogen (7N) atmosphere. The annealing process was carried out in a rapid thermal processing system (AS-Micro RTP, Annealsys, Montpellier, France). All samples were annealed for 3 min, with a temperature ramp-up time of less than 20 s. Depending on the melting temperature of the metal, various annealing temperature ranges were proposed, varying from 650 °C to 950 °C.

The surface morphology and material composition were characterized by an atomic force microscope (AFM, Veeco MultiMode with NanoScope V) and a field-emission scanning electron microscope (FE SEM, Hitachi Su6600, Tokyo, Japan) equipped with an adapter for energy-dispersive X-ray spectroscopy (EDS, Thermo Scientific UltraDry Silicon drift detectors and Thermo Scientific NORAN System 7, Waltham, MA, USA). For EDS measurements, the accelerating voltage was set to 15 kV, which exceeds the ionization energy of most X-ray emission lines of the investigated metals, ensuring sufficient overvoltage necessary for reliable measurements.

Micro-Raman measurements were carried out using a T64000 Horiba Jobin-Yvon (Kyoto, Japan) spectrometer under ambient conditions. The samples were excited with a 532 nm semiconductor laser (non-resonant excitation). The system was configured in backscattering geometry without polarization analysis. A 0.1 mm entrance slit and a single-subtractive mode provided a spectral resolution of approximately 0.5 cm^−1^. A confocal microscope with objectives of 100× magnification enabled illumination of the sample surfaces of 1 µm wide laser spot on the sample surface. The Raman signals were detected using a liquid-nitrogen-cooled Horiba Symphony II CCD camera (Kyoto, Japan), ensuring high sensitivity and low-noise acquisition.

ImageJ software (version 1.54r) was used to analyze SEM images of metal nanoislands, which allows for the determination of the number of islands, their surface area, and their roundness. Assuming a spherical particle shape, the mean particle radius was determined for particle size imaging. The sphericity of the islands can be determined using the roundness parameter, which was determined using the ratio: (island surface area)/(π * ((major axis of island)/2)^2^).

For the roundness parameter formulated in this way, its value is 1 for a perfect circle and 0 for an infinitely thin and long line. Surface coverage ratio was also calculated, to determine the extent to which the metal islands cover the surface of the sample.

## 3. Results and Discussion

The metals investigated in this study were selected based on their potential application in two key areas: as durable masks in RIE processes, and as catalytic materials in metal-assisted chemical etching (MaCE). Metals intended for use as masks in RIE processes must exhibit high resistance to plasma chemistry (specifically, chlorine-based plasma for GaN), whereas those used as catalysts in MaCE require a high work function.

Table 1 shows the properties of selected metals. A wide range of melting temperatures (from 962 °C to 2623 °C) can be seen. The solid-state dewetting process of thin metal layers can be accelerated by increasing the annealing temperature. However it was demonstrated that it is not necessary to achieve the temperature of the metal’s melting point [7]. The SSD process also depends on the type and thickness of the layer and the surface properties of the substrate. Thin films of some metals dewetting without thermal activation reveal a characteristic surface discontinuity [8]. On the other hand, the ability to self-assemble decreases significantly for layers thicker than 30 nm [11]. Therefore, the thickness of the metal layers was set at 10 nm, which represented a compromise between the size of the structures and the activation temperature of the SSD process. Assuming that refractory metals with melting points above 2000 °C will require higher activation temperatures for SSD processes, as a first step of our investigation, the thermal stability of GaN layers themselves in a nitrogen atmosphere was examined.

At high temperatures, GaN decomposes into gallium and nitrogen by dissociative sublimation (etching) or with liquid Ga droplet formation. The mechanism and intensity of this phenomenon depend on the annealing conditions, such as temperature and atmosphere, as well as the properties of the GaN layer itself, including its quality, polarity, and the growth method. Thermal stability of the Ga-polar layers in a nitrogen atmosphere has been demonstrated at the temperatures exceeding 1000 °C [23,24]. However, other studies have reported the possibility of thin near-surface layer decomposition in N_2_ atmospheres at temperatures approaching 1000 °C [25,26].

**Figure 1:**
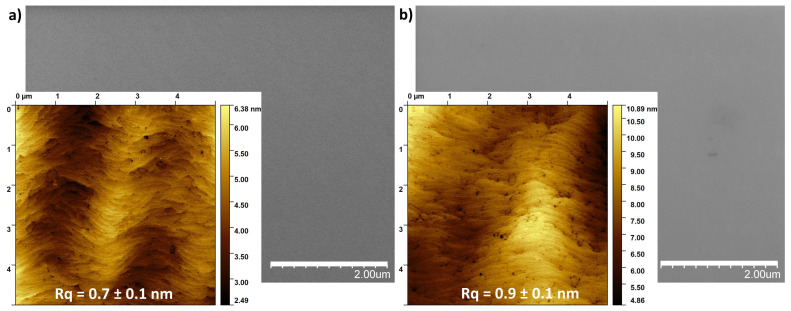
The SEM and AFM images of the GaN surface morphology from the unannealed sample (**a**) and annealed at 950 °C (**b**).

To verify the stability of our substrates, GaN/sapphire samples were subjected to RTP processes at temperatures up to 950 °C for 3 min. In Figure 1, the images of the surface morphology taken using SEM and AFM microscopes from an unannealed sample (a) and annealed at 950 °C (b) are presented. Surface characterization did not reveal any significant deterioration in its surface structure resulting from thermal decomposition of the GaN layers. No gallium droplets formation was observed on the surface. Furthermore, the surface roughness, expressed by the root mean square (RMS) parameter, did not differ significantly, measuring 0.7 ± 0.1 nm and 0.9 ± 0.1 nm for the unannealed and annealed samples, respectively (values averaged from 5 different locations). These results confirm that the GaN/sapphire substrates were thermally stable under the applied conditions, proving a reliable baseline for investigating the effects of annealing the overlying metallic films up to 950 °C.

Based on the morphological studies, the investigated metals were categorized into three groups according to their distinct behaviours at elevated temperatures: materials that underwent structural reconfiguration to form nanoislands (Au, Ag); materials that induced decomposition and degradation of the GaN surface (Pt, Ni, Ru, Mo, Cr, Ti), a behaviour similar to that previously reported for Pd [9]; materials that formed a thermally stable system with GaN (Nb, V). The behaviour of each group is described in detail in the following subsections.

### 3.1. Au, Ag

**Figure 2:**
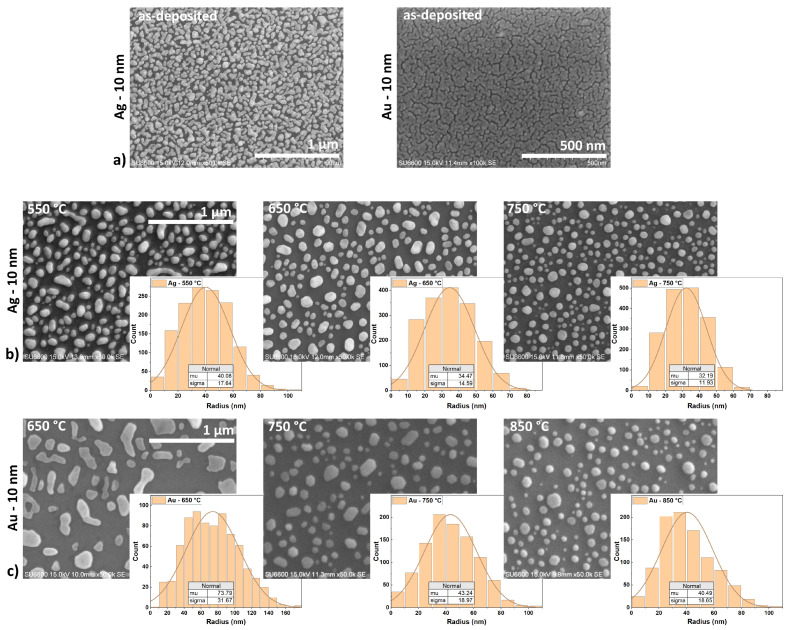
The SEM images of the as-deposited 10 nm Ag and Au layers on GaN (**a**); 10 nm Ag layer on GaN annealed at 550, 650, and 750 °C (**b**); 10 nm Au layer on GaN annealed at 650, 750, and 850 °C (**c**).

Figure 2 presents SEM images of samples with 10 nm Au and Ag layers on a GaN layer. Before the annealing process, the metal layers showed a tendency to lack wettability of the GaN substrate surface (Figure 2a). The as-deposited Au layer was discontinuous with the texture, resembling an orange peel. The as-deposited Ag layer showed significant structuring and self-organization, even before the annealing process.

The structurisation process of thin metal films is caused by the system’s tendency to minimize its total interfacial energy. This process is carried out by transporting the mass of thin metal layers by surface diffusion in order to reduce the free energy at the interfaces and obtain the equilibrium shape of the islands. For islands characterized by isotropic surface energy, the energy remains uniform over the entire plane. In this case, if the islands are located on a solid substrate, the energy balance can be described by the Young–Dupré equation [7]: $\gamma_{s}=\gamma_{is}+\gamma_{i}*cos\theta$, where $\gamma_{is}$ is the surface energy of the interface between the island and the substrate $\gamma_{s}$ is the surface energy of the substrate, $\gamma_{i}$ is the isotropic surface energy of the island, and θ is the equilibrium angle between the three phases. The occurrence of this process is more prominent for thinner layers and is even possible at room temperature (without additional annealing) for evaporated ultra-thin layers [8].

Figure 2b and Figure 2c show the effect of the annealing process of GaN substrates with Ag (b) and Au (c) layers, respectively. Silver has the lowest melting point of the tested metals. In the studies, Ag samples were annealed in the lowest temperature range. Figure 2b shows example SEM images of the sample surfaces with an Ag layer annealed at different temperatures of 550, 650, and 750 °C. As a result of thermal activation, the Ag layers self-organized and formed spherical structures in the form of separated nanoislands. For Au layers, a higher annealing temperature range of 650, 750, 850 °C was used. This was dictated by the higher melting point of gold and the research efforts to obtain spherical structures. In this case, the SSD effect also occurred, forming the round, separated islands (Figure 2c).

**Figure 3:**
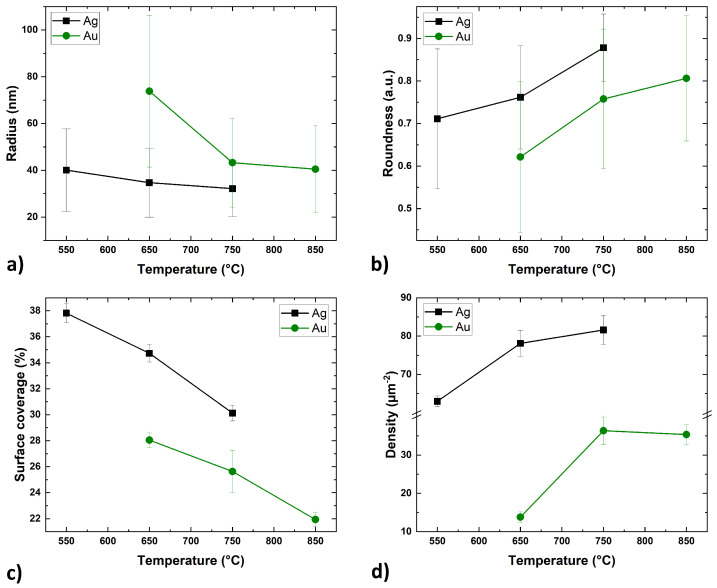
The dependence of the Ag and Au islands radius (**a**), roundness (**b**), coverage ratio (**c**), surface density (**d**), on the temperature of annealing process (annealing time—3 min).

In addition to the SEM images, Figure 2b,c, include histograms showing the size distribution of the nanostructures radius for the given metals and annealing temperatures. Table 2 summarises the quantitative results derived from SEM image analysis of the Ag and Au islands, including the mean radius with standard deviation, roundness, surface coverage, and island density. Figure 3 shows the dependence of these parameters on the annealing temperature for Ag and Au thin films.

Despite the different range of annealing temperatures, the trends of the self-assembly processes of the Ag and Au layers were similar. The size of the metal islands decreases with increasing annealing temperature (Figure 3a). At the lowest temperatures, the islands assumed irregular shapes. In the case of the Ag layer, the SSD process began during the metal deposition in the UHV system, but sufficient energy was not delivered to form isolated spherical particles. At a temperature of 550 °C, the Ag layer continued the self-assembly process, forming rounded, elongated, and separated structures. Similarly, the Au layer at 650 °C also formed separate structures, but they were less regular and rounded with a larger surface area. With the increase of the annealing temperature, the radius of the islands decreased (its natural distribution slightly narrowed (Figure 2b,c)) and the roundness coefficient increased (Figure 3b). Concurrently, the surface coverage decreased (Figure 3c), which is probably related to mass transport perpendicular to the substrate surface as the islands adopted a more spherical shape. The surface density of the nanostructures increased significantly between the first and second temperature steps before stabilizing. Providing more energy allowed for denser nucleation of voids and greater division of the layer at the same time [7]. Further increase in temperature no longer had such a significant effect on the surface density of the islands, but rather on their growth in the perpendicular axis along the surface (Figure 3d).

Overall, the Ag nanostructures on the GaN surface were consistently more rounded, while the Au nanostructures appeared to adopt more crystalline forms. Smaller islands require less energy to rebuild, and therefore they could more easily take a more spherical, equilibrium shape.

**Figure 4:**
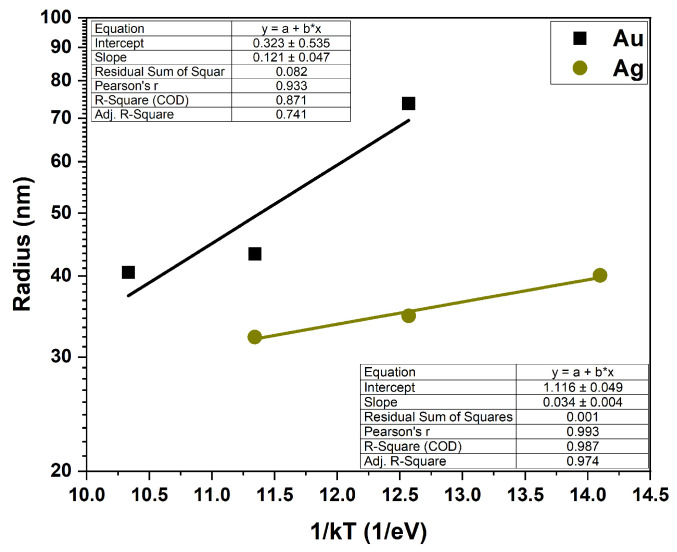
The temperature dependence of the Ag and Au islands radius—Arrhenius plots.

It is commonly accepted that surface self-diffusion is the dominant transport mechanism for solid layers. In the case of a surfaces with isotropic energies, the surface diffusion length determining the average distance of adatom migration can be written as $\lambda =\sqrt{D\tau }$, where τ is the mean residence time of the adatoms at the surface, and *D* is the surface diffusion coefficient which is given by $D=D_{0}*exp\left(E_{A}/k_{B}T\right)$, where $E_{A}$ is the energy barrier for surface diffusion, $k_{B}$ is Boltzmann constant, and *T* is the temperature [12]. A quantitative analysis for thermally activated processes (such as the SSD effect) can be carried out using the Arrhenius relationship to describe the phenomenon. Figure 4 shows the temperature dependence of the island radius (radius versus the inverse energy, 1/kT) for the Au and Ag layers, plotted on a semi-logarithmic scale. According to Arrhenius’s law, for such prepared graphs, the slopes of the lines determine the activation energy of the process which correlates with the energy of the surface diffusion barrier. The activation energy values for Au and Ag layers calculated from the lines fitted to the experimental data are 121 ± 47 meV for the 10 nm Au layer and 34 ± 4 meV for the 10 nm Ag layer.

The data for the Ag film shows a clear exponential dependence on temperature, indicating a straightforward, thermally activated process with a low activation energy. The lack of a good linear fit to the experimental data for the Au layer shows that, for low temperatures, the process is more complex and requires a much higher activation energy.

### 3.2. Pt, Ni, Ru, Mo, Cr, Ti

**Figure 5:**
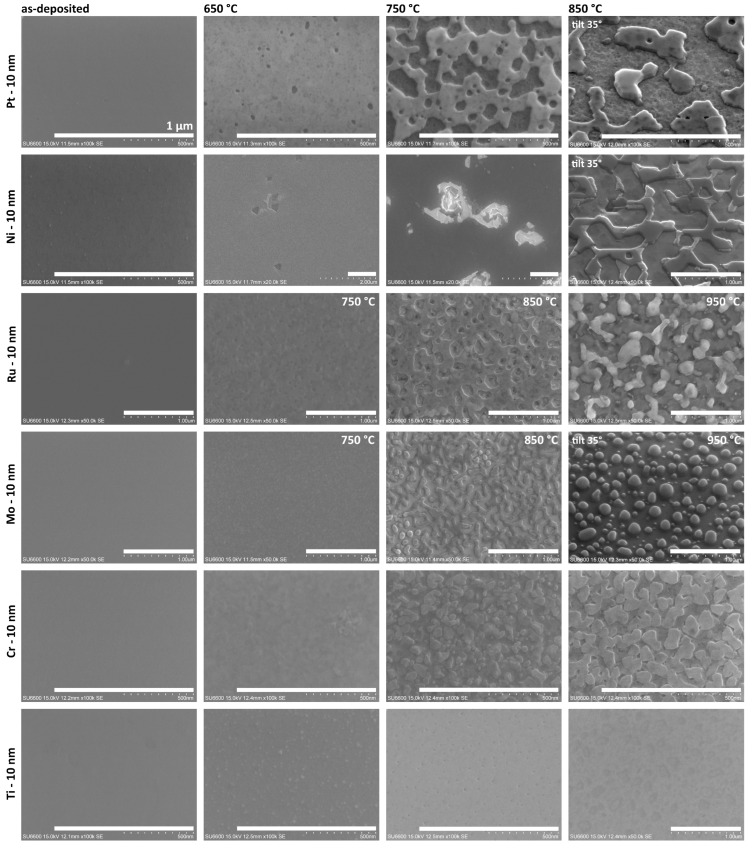
The SEM images of unannealed and annealed samples with 10 nm of Pt, Ni, Ru, Mo, Cr, and Ti layers on GaN. Scale bar—1 µm.

Figure 5 presents SEM images of 10 nm thick Pt, Ni, Ru, Mo, Cr, and Ti films on the GaN surface. The first column shows the SEM images of as-deposited metal surfaces. In contrast to Ag and Au films, these layers were continuous and smooth, with no signs of surface pre-structuring. The subsequent columns present images of the surfaces of metallic layers after annealing. The Pt, Ni, Cr, and Ti layers were processed in 650–850 °C range, while the range for Mo and Ru was increased to 750–950 °C due to their much higher melting points.

For this entire group of metals, no self-organization into spherical nanoislands, due to the SSD effect, was observed. The visible surface structuring, for annealed samples, is caused by varying degrees of degradation of the GaN layers at the metal–semiconductor interface. We have previously reported a similar degradation of GaN induced by Pd films [9]. However, the surface morphology of the metal-GaN varied, depending on the specific metal used.

Comparative studies on the SiO_2_ surface (Supplementary Materials, Figure S1) showed that for Pt and Ni layers, the SSD process fully occurred in the entire range of annealing temperatures, forming spherical islands. For Ru layers, the process occurred partially without the formation of discrete structures. The Mo and Cr layers exhibited surface structuring, whereas the Ti layer remained relatively stable on the SiO_2_ surface.

#### 3.2.1. Pt

For the Pt layer annealed at the lowest temperature of 650 °C, initial stages of void nucleation were observed (Figure 5). At higher temperatures, these voids began to coalescence and initial structuring of the GaN became evident. At 850 °C, separated, irregular, and thick island structures with sharp crystalline shapes were formed. The morphology of the entire exposed GaN surface was degraded, exhibiting significant inhomogeneity and roughness. This suggests that the degradation process at the Pt-GaN interface initiated before the metal layer underwent the self-assembly process. The situation was different in the case of Pd layers, where metal first undergoes self-assembly on an undamaged surface. Subsequently, the resulting Pd islands degrade the GaN layer in their immediate vicinity, causing pitting [9]. However, in both cases, the degradation occurred at a temperature lower than the thermal decomposition of the GaN layer. Metal layers or particles can catalyse GaN degradation, similar to how condensed Ga droplets act as nucleation sites on the GaN surface, which lowers the material’s decomposition temperature [27]. Analogous mechanisms occur during the rapid thermal processing in GaN-based device technology and are demonstrated in the improvement of ohmic contact properties and the deterioration of Schottky barrier diodes [28,29,30]. The presence of Pt or Pd as catalysts in the system decreases the activation energy, i.e., the energy needed to overcome the relatively large barrier of atomic bonds of crystalline GaN. Reducing the activation energy by the catalyst means that less energy is needed to initiate the GaN decomposition reaction. Analysing the topography observations of samples with a Pt layer compared to Pd, they were characterized by greater thermodynamics of the surface degradation process.

**Figure 6:**
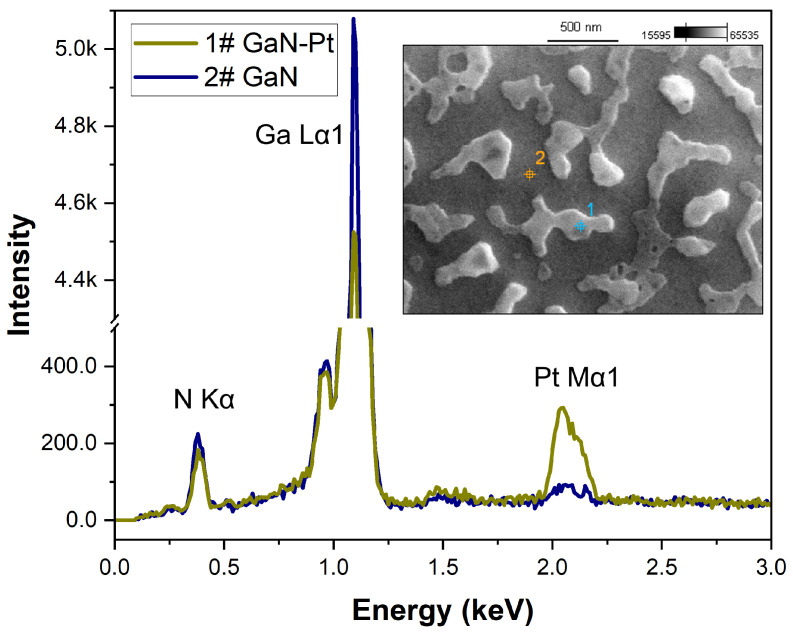
The EDS spectra of point measurement performed on a GaN-Pt sample annealed at 850 °C; the SEM image of the sample surface with marked measurement points: 1—Pt island on a GaN; 2—GaN surface between Pt islands.

In parallel with SEM imaging, microanalysis of the surface composition was performed using the EDS technique. The analysis involved point measurements performed directly on the metal islands and on the exposed GaN surface between them. An example EDS spectrum for a Pt layer annealed at 850 °C is shown in Figure 6. Table 3 summarizes the quantitative EDS results for all metals in this group at their highest respective annealing temperatures, comparing the composition of the metal islands to the exposed GaN surface.

It should be noted that EDS is a volume-sensitive technique, not purely surface sensitive. Analysis is performed from a certain volume below the surface, and the penetration depth of the electron beam depends on the beam accelerating voltage, atomic mass, atomic number, and density of the tested material [31]. Furthermore, there are no generally accepted correction models for quantitative analysis of complex composite materials containing heavy and light elements. Therefore, the EDS results presented here should be interpreted qualitatively, rather than as precise quantitative measurements.

The quantitative EDS results presented in Table 3 show that point measurement on the Pt island areas demonstrated a 2.5% higher gallium content and over 5% lower nitrogen content than the measurement taken outside these areas. This suggests that, similarly to the case of Pd layers [9], GaN decomposition occurs at the metal–semiconductor interface. Consequently, nitrogen atoms are released while gallium atoms dissolve in the Pt metal islands, forming a metal gallide. In general, the formation of ternary Pt-Ga-N or Pd-Ga-N phases is considered unlikely [32]. However, Pt and Ga atoms can form numerous intermetallic compounds, such as Ga_6_Pt, Ga_7_Pt_3_, Ga_2_Pt, Ga_3_Pt_2_, GaPt, Ga_3_Pt_5_, GaPt_2_, and GaPt_3_, via eutectic, eutectoid, and peritectic reactions, even at room temperature. According to the phase diagram of the Ga–Pt binary system, the most probable intermetallic compounds are Ga_6_Pt and Ga_7_Pt_3_, due to their eutectic temperatures of 290 °C and 822 °C, respectively. Moreover, at temperatures above 290 °C, the Ga_6_Pt intermetallic compound can transform into the Ga_7_Pt_3_ phase [33,34].

**Figure 7:**
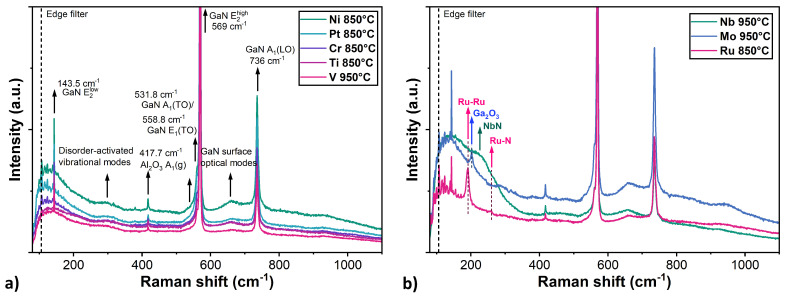
(**a**) Raman spectra of samples with GaN layers coated by Pt, Ni, Cr, Ti, and V layers and annealed at the highest investigated temperature—no clear signs of metal presence or layer decomposition. (**b**) The signal from samples with Ru, Mo, and Nb layers annealed at the highest temperature—revealing the effect of annealing and structure decomposition.

Raman spectroscopy was also employed to investigate the composition of GaN layer with metallic coatings and their possible decomposition induced by thermal annealing. For most structures, the spectra were dominated by characteristic signals originating from both the GaN layer and the Al_2_O_3_ substrate (Figure 7a). No clear signal from metallic layers or their phases was observed in samples with Pt, Ni, Cr, Ti and V layers. In particular, the following GaN-related phonon modes were observed: E_2_(low) at 143.5 cm^−1^, E_1_(high) at 568.8 cm^−1^, and A_1_(LO) at 735.6 cm^−1^. Furthermore, the asymmetric broadening of the E_2_(high) mode indicates the presence of A_1_(TO) and E_1_(TO) modes at 558.8 cm^−1^ and 531.8 cm^−1^, respectively, which suggests a slight deviation from the ideal Z(–,–)Z scattering configuration [35]. In addition, two broad features were identified: one near 300 cm^−1^, attributed to disorder-activated vibrational modes [36], and another around 650 cm^−1^, associated with GaN surface optical modes [37]. A narrow peak at 417.7 cm^−1^ corresponded to the A_1_(g) mode of the Al_2_O_3_ substrate. However, these results alone are insufficient do determine whether the GaN layers remained stable under thermal annealing. The main limitations of Raman spectroscopy in this study were: (i) the strong signal from GaN layer, which prevented detection of the weak metallic modes; (ii) the high reflectance of the metal, which hindered light penetration through the metal caps, and therefore made investigation of the structural properties at the GaN-metal interface difficult; and (iii) the poor Raman scattering of metals, which do not exhibit characteristic active vibrational modes.

#### 3.2.2. Ni

For the Ni layer annealed at 650 °C, sporadic hole formation was observed (Figure 5). Then, at a higher temperature of 750 °C, distinct craters are formed in the locations of these initial holes. This crater formation may have resulted from the decomposition of the GaN layer along the dislocation, an effect previously observed during long-term annealing of GaN/Ni samples at 500 °C [38]. However, interactions at the metal-GaN interface, such as the formation of Ga_4_Ni, are known to occur during deposition and are responsible for lowering the Schottky barrier [39]. According to the phase diagram of the Ga–Ni binary system, the most probable intermetallic compounds are Ga_5_Ni, Ga_7_Ni_3_ and Ga_4_Ni_3_ as their eutectic temperatures are below 564 °C. Studies have also shown that during the annealing of contact metallization, nitrogen atoms released from decomposed GaN can combine with the metal to form nickel nitrides, such as Ni_4_N or Ni_3_N [38,39]. These nitrides may also be responsible for the quasi-hexagonal shapes of the metal structures observed at the highest annealing temperature (Figure 5), possibly due to GaN surface reconstruction by a thin interfacial layer.

However, EDS measurements did not show significant diffusion of nitrogen atoms into the nickel layer, unlike the gallium atoms. Point measurements on the metal structures revealed a 1% higher Ga content and an almost 6% lower N content compared to the surrounding exposed surface (outside ot the island area). Furthermore, the presence of Ni-N was also not revealed by the Raman spectroscopy, which suggests that most of the nitrogen was likely volatilized from the surface as a result of the GaN decomposition.

#### 3.2.3. Ru

In the case of the Ru layer annealed at an initial temperature of 750 °C, the surface became highly developed and inhomogeneous, with numerous voids appearing (Figure 5). At the next temperature of 850 °C, the growth of these voids in the Ru layer and the formation of craters penetrating deep into the GaN layer were observed. At the highest annealing temperature of 950 °C, significant structuring and decomposition of the GaN layer surface were evident, with the separated metallic structures pitting the GaN to form surrounding craters. Ruthenium layers have been extensively studied as Schottky contacts to GaN, demonstrating relatively good stability during annealing processes and also as a barrier material that prevents interlayer diffusion in multilayer contact metallization [40,41]. However, these studies also showed that Ru layers lose their stability for these purposes above annealing temperatures of 600 °C, considered in our work. The thermal treatments in our study were carried out at much higher temperatures, where a clear lack of stability in the Ru-GaN system was observed. Furthermore, similar to platinum, ruthenium is a very active metal with many catalytic properties [42]. These properties accelerate various chemical processes and can also catalyse the decomposition of GaN. To date, a Ga–Ru binary alloy phase diagram has not been reported. However, the phases GaRu, Ga_2_Ru, and Ga_3_Ru have been investigated as narrow-band-gap semiconductors [43,44].

The EDS measurements revealed a low nitrogen content in the near-surface region both on the Ru structures and in areas outside them. This suggests, that the presence of ruthenium strongly catalysed the decomposition of the GaN layer, leading to the release of nitrogen. Point measurements on the Ru structures indicated a 3.1% higher Ga content and 6.5% lower N content compared to the measurement carried out on the outside of the island area. This implies that while nitrogen volatilises, the resulting gallium droplets combine with the ruthenium layer and in some parts probably form intermetallic compounds. In support of this, the GaN sample coated with Ru exhibited a strong signal at 191 cm^−1^ and a weak signal at 280 cm^−1^ in the Raman spectra (Figure 7b). These peaks correspond to characteristic vibrational modes of ruthenium-based structures and have been assigned to Ru–Ru stretching vibrations and Ru–N modes, respectively, which suggests that a certain ruthenium nitridation process had occurred [45].

#### 3.2.4. Mo

Molybdenum layers annealed at 750 °C were relatively stable, with only slight surface granularity observed. At the next annealing temperature of 850 °C, SEM imaging revealed the self-assembly process of Mo layers into relatively dense worm-like structures. At the highest temperature of 950 °C, the Mo layer reconfigured into discrete spherical islands with quite regular shapes (Figure 5). However, images taken at an angle show that the spherical structures stand on pedestals, which suggests surface decomposition of GaN. This decomposition appears to have facilitated the self-organisation of the island structures, a conclusion supported by the reference studies on the SiO_2_ surfaces, where the full SSD process of the Mo layer did not occur. Furthermore, this suggests that the metallic structures acted as a mask to block GaN decomposition.

The binary phase diagram of the molybdenum-gallium shows several peritectic phases, of which the most frequently mentioned are Mo_3_Ga (m.p. 1820 °C) and Mo_8_Ga_41_ (m.p. 835 °C) [46,47]. Experimental studies of GaN contacts have revealed that the β-Mo_2_N phase also occurs in the Mo-Ga-N material system at 800 °C [48]. The EDS measurement results indicated a significantly higher content of Ga atoms relative to N atoms across the entire surface, regardless of the location, which is indicative of the overall surface decomposition of GaN. Compared to the overall exposed surface, point measurements on the Mo islands revealed a 2.1% lower Ga atoms content, while the N atoms content remained at a similar level. This suggests that, in the given case, it is unlikely that intermetallic compounds form at the interface, but rather a thin nitride layer. This implies that gallium does not agglomerate under the Mo islands; instead, as a result of GaN decomposition, the material sublimates from the exposed surface.

In the Raman spectra of the sample with a Mo layer annealed at 950 °C, a narrow peak occurs at 202 cm^−1^ (Figure 7b). This feature could not be attributed to Ga–Mo or Mo–N vibrations. However, its position is in agreement with the Ga_2_O_3_ Raman mode, suggesting the GaN layer decomposition and partial oxidation of the remaining gallium [49].

#### 3.2.5. Cr

In the case of the Cr layer annealed at the lowest temperature of 650 °C, the formation of a grain structure was observed (Figure 5). At higher annealing temperatures, flat, separated structures were formed on the sample surface. However, EDS analysis could not identify areas without Cr content. Regardless of the location, point measurements showed an atomic chromium content of 1.6–2%. This suggests either insufficient separation of the structures, or the persistence of a thin chromium layer on the GaN surface, even after restructuring. Research on ternary Cr-Ga-N systems has shown that the phases condensed in thermodynamic equilibrium with GaN at 800 °C are CrN and liquid Ga. In addition, there are two transition phases, which probably form first: Cr_2_GaN and Cr_3_GaN [50,51]. From the perspective of the Ga–Cr binary phase diagram, the most probable intermetallic compounds are Cr_5_Ga_6_ and CrGa_4_, due to their eutectic temperatures of 775 °C and 700 °C, respectively. However, EDS measurements did not reveal increased gallium content in the island areas, indicating the absence of a liquid Ga phase. The difference between Ga and N content was relatively low compared to the other metal layers.

**Figure 8:**
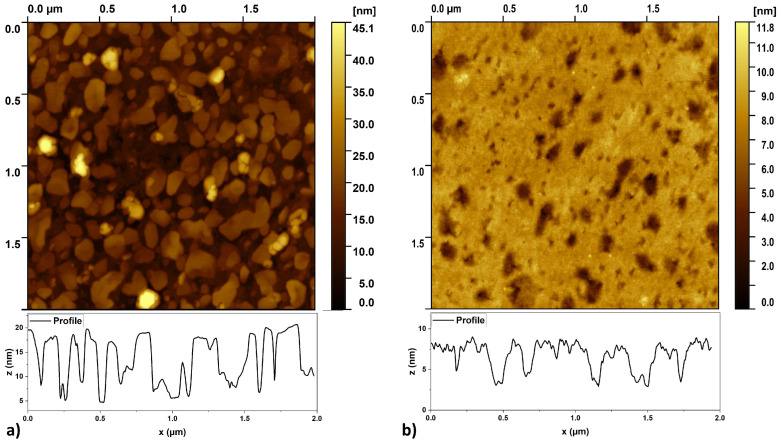
(**a**) The AFM images and example surface profiles of samples annealed at 850 °C (**a**), 10 nm Cr layer and (**b**), 10 nm Ti layer.

To further investigate the surface topography, AFM measurements were performed. Figure 8a shows an AFM scan and a representative surface profile of a sample annealed at 850 °C, revealing flat structures that are consistent with the SEM images. The height of the metallic structures was in the range of 10–15 nm, i.e., slightly more than the thickness of the Cr layers. These results suggest that significant degradation of the GaN layer did not occur, and the observed morphology is primarily due to dewetting and self-assembly process of the Cr layer. The island formation was likely facilitated by partial decomposition of GaN, given the heterogeneity of the inter-island surface observed in AFM maps. Although metal mass transfer could be inhibited by the formation of nitrides at the interface, as indicated by the relatively high nitrogen content in EDS measurements.

#### 3.2.6. Ti

The Ti layer exhibited even greater thermal stability than the Cr layer. Similar to the Cr layer, the formation of a grain structure in the layer could also be observed at the lowest annealing temperature (Figure 5). However, at higher temperatures, only small voids formed. At 850 °C, separated structures were also visible, but they were much less pronounced than those observed in the case of the Cr layer. However, surface mapping using AFM microscopy (Figure 8b) revealed that the structure features visible in the SEM images are not islands, but holes. The depth of these holes in the layer did not exceed 5–6 nm, which is less than the thickness of the layer. This value suggests that the formation of voids is not caused by a typical dewetting process, but rather by local partial melting/etching of the metal layer into GaN.

Similar to the Cr layers, EDS point measurements were unable to identify areas without Ti content. Regardless of the measurement location, the atomic content of Ti was 1.3–1.5%. This may also be due to measurement inaccuracies or a thin layer of titanium remaining on the GaN surface. Furthermore, the difference between Ga and N content was the smallest compared with other metals. This suggests that while both Cr and Ti layers undergo partial nitridation, the Cr layer induces significantly more surface structuring compared to the Ti layer.

Available literature contains numerous studies showing the formation of titanium nitride on the Ti/GaN interface during RTP processes of ohmic contacts at temperatures above 600 °C. The nitridation of the Ti layer is a result of the diffusion of N atoms from GaN. The escape of nitrogen atoms leads to the formation of N vacancies in the GaN lattice, which act as donors and increase the local carriers concentration. Thus, the formation of the TiN layer helps improve the properties of multilayer ohmic contacts where Ti is used as the initial adhesion/contact layer [52,53].

### 3.3. Nb, V

**Figure 9:**
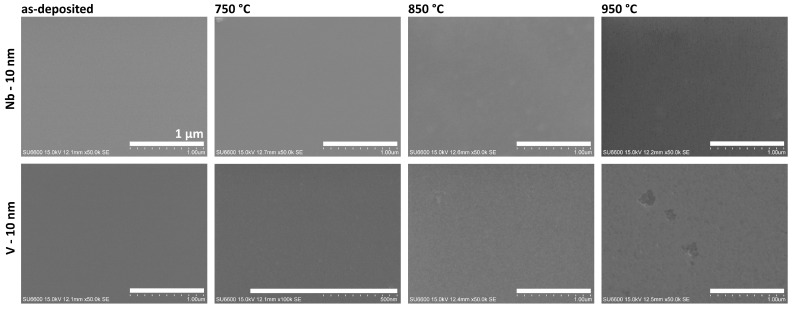
The SEM images of unannealed and annealed samples with 10 nm of Nb and V layers. Scale bar—1 µm.

The last group of metals showed high thermal stability with no significant structuring on either the GaN or SiO_2_ surfaces (Supplementary Materials, Figure S2). Figure 9 shows SEM images of samples with thin layers of Nb and V on the GaN surface, both before and after the annealing process. This group of metal layers was annealed in a higher temperature range up to 950 °C.

The niobium layer exhibited the highest stability across the entire investigated temperature range. SEM images confirm no changes in the topography or morphology, showing a smooth, homogeneous surface, with no evidence of a structuring effect in the Nb layer or degradation of the underlying GaN surface. The Ga–Nb binary phase diagram indicates that, despite the existence of intermediate phases such as GaNb_3_, Ga_2_Nb_3_, GaNb, Ga_3_Nb_2_ and Ga_3_Nb, the solubility of Ga in Nb is negligible at temperatures below 1000 °C [54]. However, XPS studies have confirmed the formation of NbNx phases in the case of GaN substrates with a thin Nb layer annealed at 850 °C [55]. For the Nb-coated GaN layer, in the Raman spectra, a broad band extending from 100 to 300 cm^−1^ was detected (Figure 8b). This result is consistent with acoustic modes reported for NbN single crystal [56], suggesting that nitrogen could release from partial GaN decomposition and formed Nb-N bonds. However, the weak Raman signal of niobium nitride could also indicate the formation of ultrathin layers of interfacial compounds at the metal-GaN interface resulting the interaction of niobium atoms with the dangling bonds of nitrogen atoms at the GaN surface [57].

Vanadium layers also demonstrated high thermal stability on the GaN surface. However, in this case, at a temperature of 850 °C, the formation of a grain structure in the metal layer could be observed. At the higher temperature of 950 °C, the continuity of the layer was interrupted by the formation of sporadic voids. AES and XRD studies conducted of ohmic contacts to GaN have shown that on the annealed V-GaN interfaces, N atoms and, to a lesser extent, Ga atoms diffuse into the V layer, forming interphase compounds such as VN, V_2_N, GaV_3_, Ga_5_V_6_, Ga_7_V_6_ [58,59], but the Ga–V binary phase diagram indicates that the most probable intermetallic compound is V_8_Ga_41_ due to its eutectic temperature of 500 °C.

### 3.4. Potential Application

The research conducted has yielded insights into application for the fabrication of self-assembled metal nanopatterns on large-format GaN layer surfaces. Thin metal layers such as Au and Ag show the ability to self-assemble into separated nanostructures on the GaN surface, without degradation of the substrate. The Pt or Ni layers may also undergo self-assemble process, but the GaN surface should be protected by the thin capping layer, e.g., SiO_2_, SiN_*x*_, AlN. This metallic nanostructures can act as a mask in RIE process. However, dielectric capping layers reduce the efficiency and selectivity of RIE processes, where chlorine plasma is mostly used for dry etching of GaN layers [60]. Additional, dielectric layers could also limit the applicability of open circuit metal-assisted chemical etching. In this process, the metal nanostructures acts as a cathode, that initiates the reduction reaction of oxidizing agent with simultaneous electron gain, and semiconductor surface acts as the anode, where the oxidation process occurs with the release of electrons. This causes an electrical potential difference and a local current flow, which results ind surface oxidation and subsequent etching of the material in acid solution [61]. The cap layer could reduce the current flow, decreasing etching efficiency. The knowledge about the possibility of self-assembled metal nanostructures on GaN surface is poised to catalyse the development of novel, cost-effective technologies for AIIIN layer nanostructuring through the utilisation of wet or dry etching methods.

Metals that exhibit thermal stability on the GaN surface, such V or Nb, can be applied as masking layers or barrier layer for stopping interdiffusion in high-temperature GaN processing like selective area epitaxy or RTA process of metallic contacts. Layers with high thermal stability of the surface morphology on GaN can also be used as various types of electrodes (reference electrode or electrodes in MIM capacitors) in high-temperature power devices.

When considering high-temperature processing in GaN-based device technologies, attention should be focused on metals such as Pt, Ni, and Ru, which can strongly catalyze GaN surface degradation.

## 4. Conclusions

In this paper, we present the results of studies on the thermal stability of thin metal layer morphology on GaN surfaces. Among the wide range of metals investigated, only Ag and Au films demonstrated the ability to self-assemble into separate spherical islands without significant degradation of the GaN surface. The SSD process for Ag layers occurred much faster and at lower temperatures, with a calculated activation energy of 30 meV compared to 170 meV for the Au layer (within the investigated temperature range). The Ag island structures were smaller, more spherical, and exhibited higher areal density than those formed from Au.

A second group, including Pt, Ni, and Ru layers, exhibited strong catalytic properties, including the degradation of GaN surfaces at temperatures above 600 °C. As a result of GaN decomposition, significant surface structuring occurs and gallium atoms dissolve in the metal islands. In this situation, the exposed GaN surface is degraded, heterogeneous, with numerous defects, pits, and irregularities. For Mo layers, the annealing process led to the formation of spherical structures, but studies indicated that GaN decomposition was also responsible for initiating the SSD process. As a result, thermal etching of GaN layers was observed through material sublimation, where Mo islands acted as masks. The Cr and Ti layers exhibited characteristics of GaN surface structuring after annealing processes, but no significant degradation of the GaN surface occurred. In the case of the Cr layer, a self-assembly process occurred, probably assisted by partial surface decomposition of GaN. The structuring of the Ti layer consisted in forming craters as a result of metal local melting into GaN.

The third group, V and Nb, demonstrated high thermal stability on the GaN layer, maintaining their morphological integrity. In the case of the Nb layers, the exceptional stability was likely enhanced by the formation of Nb-N bonds at the metal-GaN interface.

## Figures and Tables

**Table 1:** Metal type, the measured layer thickness and deposition rate, properties of the metals: melting point and work function of the metal.

Metal	Thickness (nm)	Rate (Å/s)	T_*m*_ (°C)	WF (eV)	Annealing Effect on GaN/SiO_2_
Au	10.7 ± 0.2	1.1	1064	5.1–5.47	SSD/SSD
Ag	10 ± 0.6	0.6	962	4.26–4.74	SSD/SSD
Pt	7 ± 0.1	0.4	1768	5.12–5.93	deg ^1^/SSD
Ni	8.7 ± 0.5	1	1455	5.04–5.35	deg ^1^/SSD
Ru	11.2 ± 0.2	0.03	2334	4.71	deg ^1^/stru ^1^
Mo	10.2 ± 0.4	0.6	2477	3.95–4.87	deg ^1^/none
Cr	8 ± 0.7	1	1907	4.5	deg ^1^/none
Ti	10 ± 0.5	1	1668	4.33	deg ^1^/none
Nb	11.9 ± 0.7	0.2	2623	4.36–4.95	none/none
V	11.5 ± 0.5	0.5	1910	4.3	none/none

^1^ deg—GaN surface degradation; stru—metal surface structuring.

**Table 2:** The results of SEM analysis of the Ag and Au; the mean values and standard deviation of the radius, roundness, surface coverage ratio, and surface density.

Metal	Temperature (°C)	Radius (nm)	Roundness (a.u.)	Surface Coverage (%)	Density (µm^−2^)
Ag	550	40.9 ± 17.6	0.71 ± 0.16	37.8 ± 0.74	63.0 ± 1.4
	650	34.7 ± 14.8	0.76 ± 0.12	34.7 ± 0.67	78.1 ± 3.3
	750	32.2 ± 11.9	0.88 ± 0.08	30.1 ± 0.59	81.6 ± 3.7
Au	650	73.8 ± 32.4	0.62 ± 0.18	28.0 ± 0.55	13.8 ± 1.4
	750	43.2 ± 19.0	0.76 ± 0.16	25.6 ± 1.63	36.3 ± 3.6
	850	40.5 ± 18.6	0.80 ± 0.15	21.9 ± 0.55	35.3 ± 2.6

**Table 3:** The quantification results of EDS microanalysis performed on metal islands and on the GaN surface between islands for the highest annealing temperatures, atomic percentage composition of elements.

Metal-GaN	Metal (at. %)	Err (at. %)	Ga (at. %)	Err (at. %)	N (at. %)	Err (at. %)
Pt-GaN	2.7	0.1	59.6	0.3	37.7	1.4
GaN	-	-	57.1	0.4	42.9	1.3
Ni-GaN	4.9	0.2	59.2	0.4	35.9	1.2
GaN	-	-	58.2	0.3	41.8	1.4
Ru-GaN	3.5	0.1	64.6	0.4	31.9	1.4
GaN	-	-	61.6	0.4	38.4	1.3
Mo-GaN	2.3	0.1	61.2	0.4	36.5	1.3
Mo	-	-	63.3	0.4	36.7	1.3
Cr-GaN	1.6–2.0	0.1	55.4–56.0	0.3	42.5–42.6	1.3
Ti-GaN	1.3–1.5	0.1	53.2–53.4	0.3	45.3–45.4	0.5

## Data Availability

The raw data supporting the conclusions of this article will be made available by the authors upon request.

## Supplementary Materials

The following supporting information can be downloaded at https://www.mdpi.com/article/10.3390/nano15231789/s1.

## Abbreviations

The following abbreviations are used in this manuscript:

AFM	Atomic Force Microscopy
MaCE	Metal-assisted Chemical Etching
MOVPE	Metal–Organic Vapour Phase Epitaxy
EDS	Energy-Dispersive X-ray Spectroscopy
RIE	Reactive Ion Etching
SEM	Scanning Electron Microscope
SSD	Solid-State Dewetting
