# Supplementary figures and images for: Thermal Stability of Thin Metal Films on GaN Surfaces: Morphology and Nanostructuring
Source: Nanomaterials (Basel). 2025 Nov 27;15(23):1789. doi: 10.3390/nano15231789 (PMC12693282; doi:10.3390/nano15231789)

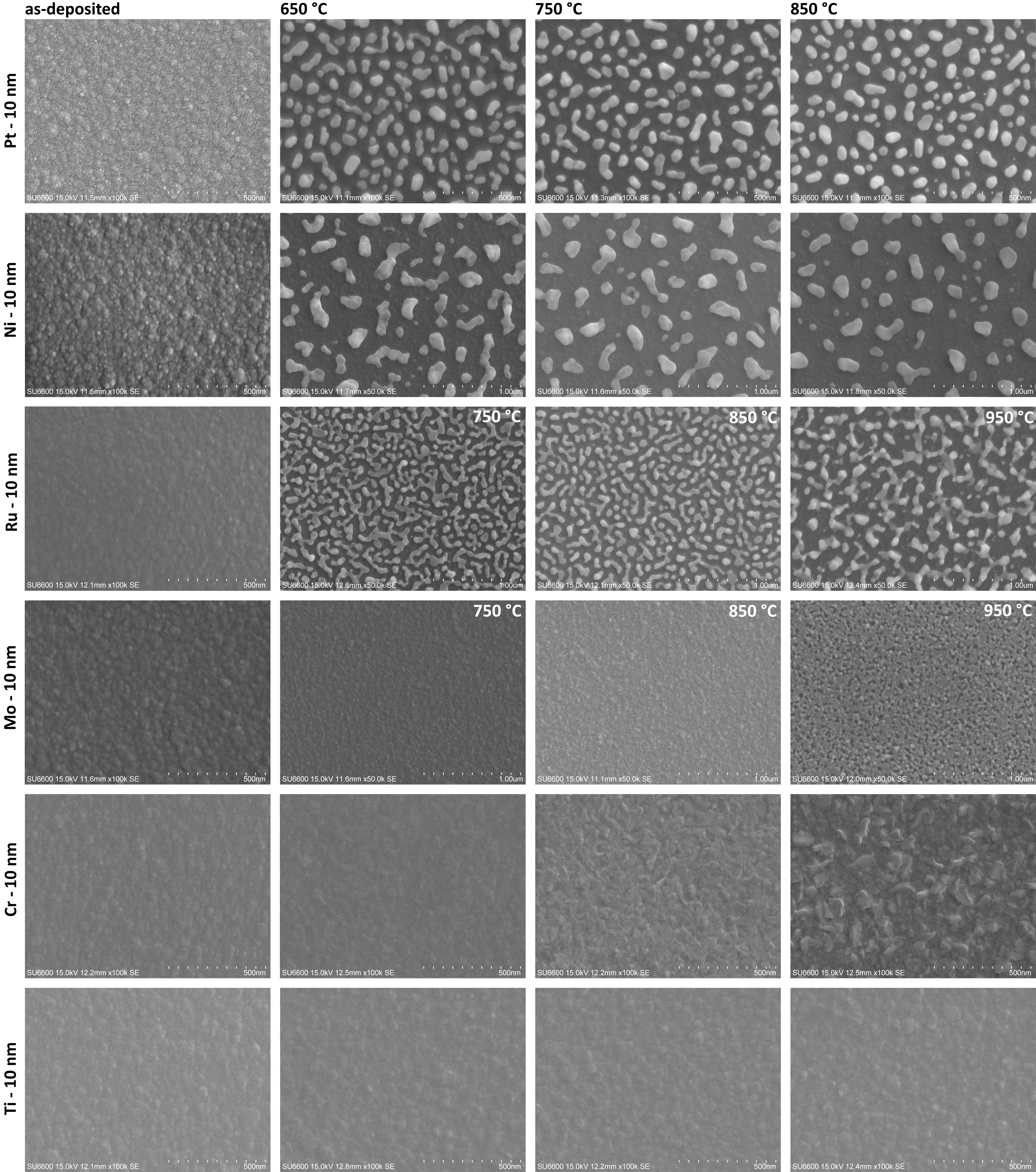

Supplement: Supplementary file 1 [file nanomaterials-15-01789-s001.zip › Figure S1.png]

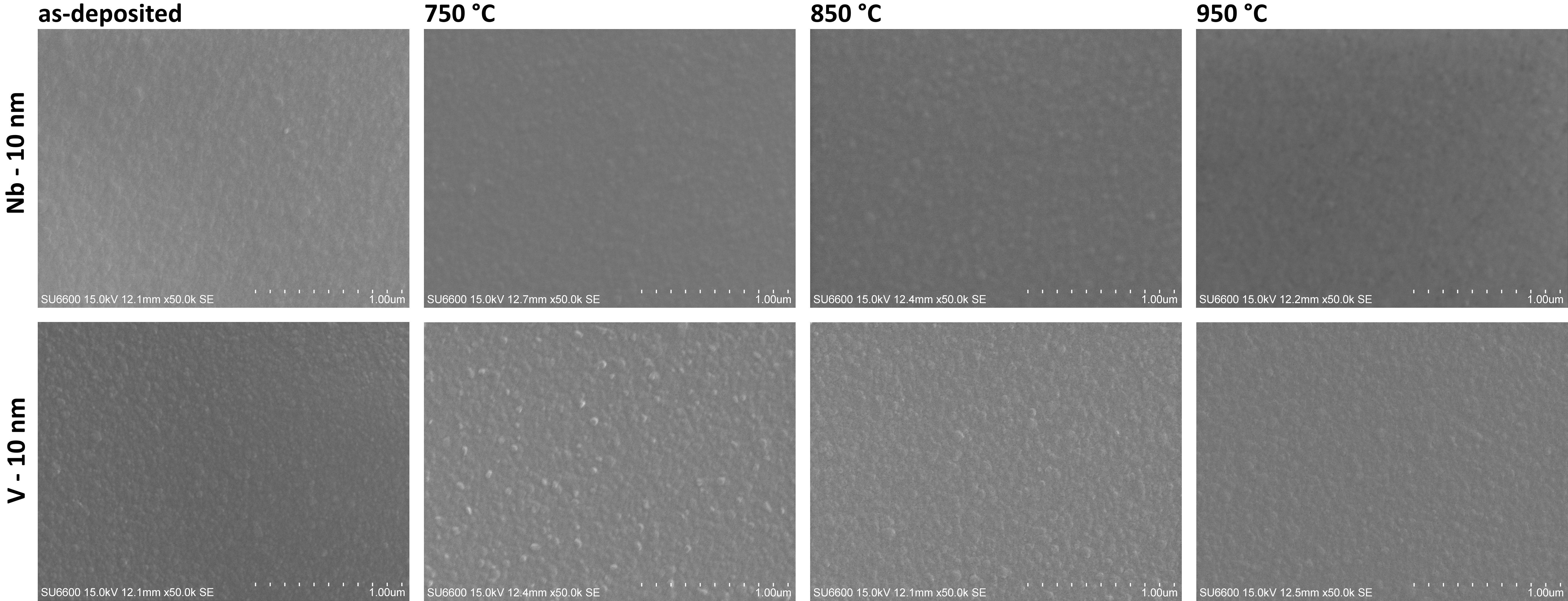

Supplement: Supplementary file 1 [file nanomaterials-15-01789-s001.zip › Figure S2.png]
